# Taurine protection attenuates bisphenol-A-induced behavioral, neurochemical, and histopathological alterations in male rats

**DOI:** 10.1007/s00210-024-03767-4

**Published:** 2025-01-17

**Authors:** Mohamed M. Nazmy, Neveen A. Noor, Faten F. Mohammed, Yasser A. Khadrawy, Nasr M. Radwan

**Affiliations:** 1 Molecular Biology and Biotechnology Department, School of Biotechnology, Badr University, Badr City, Cairo, Egypt; 2 Zoology Department, Faculty of Science, Cairo University, Giza, Egypt; 3 Faculty of Veterinary Medicine, Cairo University, Giza, Egypt; 4 Department of Pathology, College of Veterinary Medicine, King Faisal University, 31982 Al-Ahsa, Saudi Arabia; 5 Medical Physiology Department, Clinical Studies and Medical Research Institute, National Research Centre, Giza, Egypt

**Keywords:** Bisphenol-A, Taurine, Monoamines, Apoptosis, Memory, Motor activity

## Abstract

Due to the continuous exposure to bisphenol-A (BPA), the current study was conducted to evaluate taurine’s neuroprotective action against BPA’s adverse effect on the brain. Rats were grouped into control, BPA-treated rats, and taurine + BPA-treated rats. At the end of the 35-day treatment period, the memory of the rats was evaluated using the novel object test and the Y-maze test. An open-field test was used to measure motor activity. The changes in monoamines, monoamine oxidase (MAO), acetylcholinesterase (AChE), Na^+^,K^+^,ATPase, oxidative stress, caspase-3, and histopathology were evaluated in the cortical and hippocampal tissues of all groups. Data analysis by ANOVA revealed that BPA treatment induced motor hyperactivity and short- and long-term memory impairment. In the cortex, BPA decreased serotonin (5-HT), norepinephrine (NE), MAO, Na^+^,K^+^,ATPase, and nitric oxide (NO) and increased dopamine (DA), AChE, lipid peroxidation (MDA), glutathione (GSH), and caspase-3. In the hippocampus, BPA increased 5-HT, DA, NE, MAO, AChE, MDA, NO, GSH, and caspase-3 and decreased Na^+^,K^+^,ATPase. These neurochemical changes were accompanied by significant histopathological alterations. Taurine treatment prevented memory impairment and motor hyperactivity induced by BPA. Taurine attenuated the neurochemical changes, oxidative stress, and caspase-3 level. Taurine improved the histopathological change induced by BPA. In conclusion, taurine significantly prevented BPA-induced cognitive deficits, motor coordination impairments, neurotransmitter imbalances, histopathological alterations, oxidative stress, and apoptosis.

## Introduction

Bisphenol A (BPA) is a monomer mostly used in the manufacture of epoxy resins and polycarbonate plastics. Globally, BPA represents one of the most widely formed synthetic compounds with an annual production of over 3.8 million tons and an atmospheric release of 100 tons (Vandenberg et al. 2010; Michałowicz 2014). It is found in the air, water, and soil (Kang et al. 2007). Although the environment is one source of human exposure to BPA, food is considered the primary exposure source as significant BPA levels were detected in canned foods (Cao et al. 2010).

BPA is a xenoestrogen, a synthetic chemical that mimics natural estrogens and can bind to estrogen receptors, acting as an endocrine-disruptor, interfering with the endocrine system, through its selective binding with the estrogen receptors (ERs) ERα and ERβ causing biological activities similar to those of estradiol (Degen and Bolt 2000).

Several adverse effects have been reported due to BPA exposure, including reduced daily production of sperm and fertility in males (Wisniewski et al. 2015), changes in the development of female offspring mammary glands (Ji et al. 2023) and fetuses (Vandenberg et al. 2007), and disturbance in the metabolism of male offspring exposed prenatally to BPA (Cabaton et al. 2013). Furthermore, BPA can cause neurotoxicity by precipitating oxidative stress, producing autophagy and apoptosis, suppressing neurogenesis, and decreasing synaptic plasticity (Santoro et al. 2019). Additionally, it has been reported that BPA induced structural and functional changes in the hippocampus and cerebral cortex, reducing hippocampal myelination potential (Tiwari et al. 2015a) as well as the survival and development of oligodendrocyte progenitor cells (Tiwari et al. 2015b). Exposure to low and high doses of BPA increased hippocampal glutamate, glutamate/GABA, and acetylcholine levels and decreased 5-HT level in male rats, suggesting a disturbance in the homeostasis of different neurotransmitters (Zhang et al. 2019).

BPA is excreted mostly in urine as glucuronide/sulfate conjugates. It occurs mostly within 24 h and has an estimated biological half-life of approximately 6 h (Thayer et al. 2015; Ramadan et al. 2020). However, the small part that is not excreted can be found in other biological fluids in lower concentration. Geens et al. (2012a, b) detected BPA in the brain (0.91 ng/g) in samples taken in 2002 during the autopsy of 11 patients at the University Hospital of Antwerp (Geens et al. 2012a). Furthermore, in a more detailed investigation, Kim et al. (2004) analyzed the brains of Nulliparous Fischer 344 female rats administering an oral dose of 100 mg/kg BPA and detected BPA accumulation after 48 h in plasma, pituitary, hypothalamus, brain stem, cerebellum, frontal cortex, hippocampus, and caudate nucleus.

Early studies showed that the prefrontal cortical and hippocampal neurons were among the main targets of BPA (Hajszan et al., 2010). Due to their importance in cognitive functions, different studies emphasized on the effects of BPA on the prefrontal cortex with respect to the start time, doses, and period of BPA exposure (Leranth et al. 2008; Eilam-Stock et al. 2012). Epidemiological studies reported a link between prenatal exposure to BPA and harmful effects on the behaviors and cognitive performance of young children, especially on working memory, executive function, and social responses (Braun et al. 2017). It has been observed that BPA results in a decrease in the density of the hippocampal dendritic spine and memory impairment (Eilam-Stock et al. 2012). Several recent studies have emphasized the requirement of exogenous interventions to reduce the adverse impact of environmental BPA exposure on some CNS functions, such as brain development and physiology after interfering with endogenous defense mechanisms (Santoro et al. 2019; Cai et al. 2020; Di Pietro et al. 2020). However, other studies reported that BPA prenatal exposure had no significant association with effects on cognitive functions (Bornehag et al. 2021). In addition, Aiba et al. (2018) reported that BPA exposure in the fetal stage did not exhibit any significant effect on hippocampal DNA methylation. Controversial data have also been published by several authors reporting both anxiolytic (Tian et al. 2010) or lack of BPA effects on anxiety and depressive-like behaviors (Fujimoto et al. 2013). These discrepancies can be explained by methodological differences between study groups.

Taurine is an amino acid containing sulfur; it is found in high content in the plasma and tissues of mammals. It is essential for several basic biological activities, including immune system function, membrane stabilization, glucose management, calcium modulation, CNS and retinal development, antioxidant activity, and reproduction (Schuller-Levis and Park 2003; Schuller-Levis et al. 2009; Schaffer et al. 2010). Taurine is regarded as a trophic factor for brain development, stimulating brain cell proliferation, and has a protective role against damages induced by toxic substances (Pasantes-Morales and Hernandez-Benitez 2010). It has a therapeutic effect against several neurological disorders (Menzie et al. 2014), such as Huntington’s disease, Alzheimer’s disease, and Parkinson’s disease. Del Olmo et al. (2004) demonstrated that taurine administration induced long-term potentiation in hippocampal synapsis after its uptake by taurine transporters. Taurine has a cytoprotective effect due to its antioxidant activity which is mediated by improving mitochondrial function through the stabilization of the electron transport chain and inhibition of reactive oxygen species production (Jong et al. 2012; Baliou et al. 2021). Recently it has been reported that taurine ameliorated the cortical structural changes induced by BPA in rats (Kandeel et al. 2024). Moreover, taurine showed a significant prophylactic effect against BPA-induced oxidative stress, neuronal pyknosis, and chromatin condensation in zebrafish brain (Pradhan et al., 2021).

The present study was carried out to investigate the neuroprotective effect of taurine against the behavioral, neurochemical, and histopathological alterations induced by BPA in male albino rats. This could be achieved by measuring the changes in motor activity, memory functions monoamine neurotransmitters, oxidative stress parameters, caspase-3, and histopathology.

## Material and methods

Adult male Wistar albino rats (3–4 months old) weighing 160–180 g were used. Wistar rat is the most popular rat strain used as experimental material in epidemiological research, clinical trial, genetic study, or even mechanism elaboration of pathway signaling. All experimental procedures were carried out in the UResearch Animal Facility (URAF) and adhered to the ethical guidelines of Cairo University Institutional Animal Care and Use Committee (IACUC), Egypt (approval no. CU/I/F/10/23). The rats were left without any treatment for 7 days to adapt to the animal house. During this period, rats were observed to ensure their health and that their behaviors were stable before experimental procedures began. They were allowed free access to standard rodent food pellets (Agricultural-Industrial Integration Company, Giza, Egypt) and tap water ad libitum. The animals were divided into polyacrylic cages (6 rats per cage) supplied with bedding materials, sawdust, nesting material (Kleenex tissues), and two wooden cubes. The bedding materials were changed regularly to maintain a clean environment. The temperature was maintained at 22–25 °C, with humidity 50–60%, and a 12:12-h light:dark period was adjusted.

### Chemicals

BPA (CAS 80-0.5-7) pure powder was obtained from the Sigma-Aldrich company (Missouri, USA). It was prepared as a suspension in distilled water. BPA suspension was prepared daily by dissolving 200 mg of BPA in 20 ml distilled water (10 mg/ml). Sulfanilamide (CAS 63–74-1), N-1-naphthylethylenediamine (CAS 1465-25-4), absolute ethyl alcohol, triethylamine, perchloric acid, trichloroacetic acid (CAS 76-03-9), and thiobarbituric acid (CAS 504-17-6**)** were purchased from the Sigma-Aldrich company. Additionally, 5,5′-Dithiobis (2-nitrobenzoicacid) (DTNB) (CAS69-78-3**)**, glutathione, acetylthiocholine iodide (CAS 1866-15-5), ethylenediaminetetraacetic acid (CAS 60-00-4), and phosphate buffers were purchased from Sigma-Aldrich. Taurine (CAS 107—35-7), supplied by Sigma (USA), was prepared daily by dissolving 400 mg of taurine in 10 ml distilled water (40 mg/ml).

### Experimental design

Thirty-six rats were divided into a control group administered orally with distilled water two times with 1 hour in between, BPA-treated group which received orally distilled water followed by BPA (25 mg/kg) with 1 hour in between (Khadrawy et al. 2016), and the protected group that received orally taurine (100 mg/kg) (Jangra et al. 2024) followed by BPA (25 mg/kg) with 1 hour in-between. All treatments were given 6 days per week for 6 weeks. All groups received the treatments two times to ensure equal handling of all groups to minimize stress or behavioral differences due to handling rather than the treatment itself. In addition, all animals were maintained under the same surrounding environmental conditions to avoid extraneous variables. At the end of the treatment period, the behavioral tests were performed. Behavioral tests were performed 3 days before decapitation. The habituation phase and the training phase were carried out on the two successive days before the last treatment. However, behavioral testing was performed immediately after the last treatment to capture potential changes in memory and cognition resulting from the treatments.

### Behavioral tests

#### Y-maze test

This test is used to evaluate the short-term memory (Luszczki et al. 2005). The apparatus of the Y-maze consists of three arms making a Y shape. These arms extend from a central platform. The dimensions of each arm are 35 cm in length, 10 cm in width, and 25 cm in height. The angle between each arm is 120°. The idea of this test depends on the normal tendency of rodents to discover a new arm rather than the familiar one. The Y-maze test was performed on two sequential days. The first day was specified for training that was performed by placing each rat at the central platform and allowing it to move freely through the maze for 8 min. On the test day, the sequence of arms entered by each rat was recorded throughout the 8-min session. Between each two animals, the maze was whipped with 70% ethanol to clean any olfactory cues that may cause errors in the observations. An actual alternation was defined as successive entries into all three arms called overlapping triplet sets. Possible alternations were identified as the total number of arm entries. The percentage of spontaneous alternation behavior was calculated using the formula of Kraeuter et al. (2018):

$$\%\;Alternation=\left(no.\;of\;alternation\;/\left[no.of\;entries-2\right]\right)\times100.$$

#### Novel object recognition test

Novel object recognition test (NOR) measures long-term memory (LTM) and cognition (Nalivaeva et al. 2012). This test depends on the innate preference of rats to explore a novel object more than an older one (Ennaceur 2010). The NOR test involves three phases carried out on three successive days. The first phase is the habituation phase, during which rats were allowed to adapt to the surrounding wooden box (30 × 30 × 30), each rat for 10 min. The second day was the training phase in which rats were allowed to familiarize themselves with two wooden objects of non-toxic materials having the same size, shape, and color. The objects were placed in opposite corners 2 cm away from the walls inside the box. Each rat was placed in the wooden box for 10 min to get acquainted with the identical objects. The test phase was performed on the third day in which one of the two identical objects was removed, and a novel object having a different size, shape, and color was placed instead. During this phase, each rat was allowed 5 min to explore the two different objects. The arena and objects were thoroughly cleaned after each rat using 70% ethanol to neutralize the odor cues. The recognition index (*RI*) was calculated as the time spent by each rat to explore the novel object (*N*) as a percentage of the total exploration time taken by the rat to explore both novel and familiar (*N*+*F*) objects (Nalivaeva et al. 2012).

$$Recognition\;index\;\left(RI\right)=\frac{Time\;spent\;exploring\;the\;novel\;object\;\left(N\right)}{Total\;exploration\;time\;\left(N+F\right)}\times100$$

The discrimination ratio (DR) is calculated as the difference between the time spent by the rat exploring the novel (*N*) and familiar (*F*) objects divided by the total time spent by the rat exploring both objects. This record ranges between +1 and −1 where a positive score indicates more time spent with the novel object, whereas a negative score expresses more time spent with the familiar object, and a zero score represents a null preference (Castillo-Carranza et al. 2015).

$$The\;discrimination\;ratio\;\left(DR\right)=\frac{Time\;difference\;between\;exploring\;novel\;and\;familiar\;objects\;\left(N-F\right)}{Total\;exploration\;time\;\left(N+F\right)}$$

#### The open field test

Open field test estimates rat motor activity. In this test, each animal is put in the apparatus for 10 min. The apparatus consists of white plywood of 72 × 72 cm and 36 cm walls. To observe the rats in the apparatus, one of the four walls was made of clear plexiglass. The floor of the apparatus was also made of clear plexiglass placed above a sheet divided into 16 squares of 18 × 18 cm. In the middle of the apparatus, a central square of 18 × 18 cm was drawn. During the habituation phase on the two successive days before performing the test, each rat was placed in the open field for 10 min to reduce anxiety-related behaviors. The measured OFT parameters are the duration of the central square (the time spent by each rat in the central square), the number of crossed lines (the number of grid lines crossed by all four paws of the rat), and the number of rearing (the number of times the rat spent standing on its hind legs) and freezing time (times the rat was completely motionless). These parameters were tracked by the SMART Video tracking system (Panlab)-Harvard. After each session, the open field apparatus was cleaned with 70% ethyl alcohol to avoid smell clues (Brown et al. 1999). OFT was applied once for each rat.

### Neurochemical measurements

Rats were sacrificed by sudden decapitation. The brain of each animal was quickly taken off the skull and placed on an ice-cold Petri dish where it was divided into two longitudinal halves. The cerebral cortex and hippocampus of each half were dissected, weighed, and frozen at − 80 °C until analyzed. The brain regions of the left half were used to measure monoamine neurotransmitters. The right half regions were used to determine oxidative stress parameters, caspase-3, and enzyme activities.

Samples from right brain tissues were homogenized in Tris–HCl buffer (pH 7.4), centrifuged at 5000 rpm and 4 °C for 10 min, and then the supernatant was stored at − 80 °C until analysis. Neurochemical analyses were performed after 7 days of sample collection.

### Determination of monoamines

The left cortex or hippocampus was homogenized in acidified n-butanol by tissue sonicator and centrifuged at 3000 rpm for 5 min at 4 °C. In a tube containing 5 ml heptane and 1.6 acetic acid (0.2N), 2.5 ml of the supernatant was added. The mixture was vortexed for 30 s and centrifuged at 3000 rpm for 5 min to separate the aqueous layer from the organic layer that was discarded. The aqueous layer was used to measure 5-HT, NE, and DA.

Then, 1 ml of the aqueous phase was used to determine NE and DA by adding 0.2 ml EDTA, 0.2 ml of alkaline sulfite, 0.1 ml of 0.1 N iodine, and 0.2 ml 5 N acetic acid. Then, the tubes were placed in a boiling water bath for 2 min, cooled using tap water, and read for NE fluorescence. Excitation was made at wavelength 380 nm, and emission was measured at wavelength 460 nm. All solutions were returned to their original test tubes, re-heated in a boiling water bath for an additional 5 min, and cooled under tap water. For DA, excitation and emission wavelengths were 320 and 375 nm, respectively.

For 5-HT determination, 200 µl of the aqueous phase of each sample was also transferred to tubes containing 1.2 ml of O-phthalaldehyde (4 mg%) (4 mg of O-phthalaldehyde were dissolved in 100 ml of 10 N HCl). The tubes were placed in a boiling water bath for 10 min, cooled using tap water, and read in a spectrofluorometer (excitation was 355 nm, and emission was 470 nm). The quantitative determination of 5-HT, NE, and DA levels was carried out according to the method of Ciarlone (1978) using a spectrofluorometer (Jasco FP- 6500, JASCO Ltd., Tokyo, Japan) supplied with a xenon arc lamp source 150 W having an excitation slit bandwidth of excitation monochromator and emission slit bandwidth of emission monochromator of 5 nm each.

### Determination of oxidative stress parameters

#### Lipid peroxidation (MDA)

Lipid peroxidation was quantified in the cortex and hippocampus of each rat by measuring the malondialdehyde level which is one of the main products of lipid peroxidation based on the method of Ruiz-Larrea et al. (1994). The formed MDA interacts with the thiobarbituric acid to give a pink color that was read spectrophotometrically at 532 nm.

#### Nitric oxide (NO)

Nitric oxide is an unstable gas with a short half-life. It is easily oxidized forming nitrite that was used to evaluate the level of NO in the cortex or hippocampus according to the method of Moshage et al. (1995). Nitrite reacts with Griess reagent forming a deep purple azo whose absorbance was measured spectrophotometrically at 450 nm.

#### Reduced glutathione (GSH)

GSH determination in the cortical and hippocampal tissue is based on reducing Ellman reagent (DTNB) by the sulfhydryl group (-SH) of GSH producing 2-nitro-s-mercaptobenzoic acid, whose absorbance can be detected spectrophotometrically at 412 nm. When GSH reacts with DTNB, mixed disulfides (GS-S-DTNB) are formed, where the thiol group replaces one of the nitrobenzoic acid moieties in DTNB. The release of the nitrobenzoic acid group as nitro-2-thiobenzoate anion (TNB −) creates a yellow anion that can be measured spectrophotometrically at a wavelength of 412 nm. (Ellman 1959).

### Enzyme activities

#### Acetylcholinesterase activity (AChE)

The cortical or hippocampal AChE activity was evaluated by the method of Gorun et al. (1978). In this method, AChE hydrolyzes acetylthiocholine iodide forming thiocholine which reduces the –SH reagent DTNB producing thionitrobenzoic acid with a yellow color whose absorption is read spectrophotometrically at 412 nm.

#### Monoamine oxidase activity (MAO)

MAO activity was estimated in the cortex and hippocampus depending on the method of Holt et al. (1997). In this method, MAO metabolizes benzylamine into benzaldehyde, whose absorbance was measured at 280 nm.

#### Na^+^,K^+^,ATPase activity

The enzymatic activity of Na^+^/K^+^,ATPase was evaluated spectrophotometrically as the difference between total ATPase activity (Na^+^/K^+^,ATPase and Mg,ATPase activity) and Mg,ATPase activity according to the method described by Tsakiris et al. (2000).

#### Caspase-3 level

Caspase level was evaluated by ELISA kit (Enzyme-linked Immunosorbent Assay Kit) for Caspase 3 (CASP3) no. (SEA626Ra 96 Tests). The microplate in this kit has been pre-coated with a CASP3-specific antibody. Standards or samples are added to the microplate wells having a biotin-conjugated CASP3-specific antibody. Next, the addition and incubation of Avidin conjugated to horseradish peroxidase (HRP) to each microplate well were carried out. Only wells containing CASP3, biotin-conjugated antibody, and enzyme-conjugated Avidin will show a change in color after TMB substrate addition. The reaction is then terminated by sulfuric acid, and the color is measured spectrophotometrically at a wavelength of 450 nm. CASP3 concentration is then determined by comparing the O.D. of the samples to the standard curve.

### Histopathological examination

Brain specimens were fixed in 10% neutral buffered formalin. This was followed by dehydration in ascending ethyl alcohol concentrations, clearing in xylene, and embedding in paraffin. The tissue was sectioned and stained by H&E (Suvarna et al. 2019). Tissue sections were examined with an Olympus BX43 light microscope and captured by an Olympus DP27 camera linked to the cellsens dimension software.

### Statistical analysis

The data obtained were expressed as mean ± SEM. Analysis of statistical significance between groups was performed by one-way analysis of variance (ANOVA) using the Statistical Package for Social Sciences (SPSS) program, version 16. When *p*-value < 0.05 was obtained, the difference was considered significant, and Duncan was carried out to compare significance between groups. The percentage difference (% difference) with respect to the control group was then calculated according to the following equation:

$$\%\;difference=\frac{Treated\;value-Control\;value}{Control\;value}\times100$$

## Results

### Behavioral data

#### Data of the novel object recognition test

**Fig. 1:**
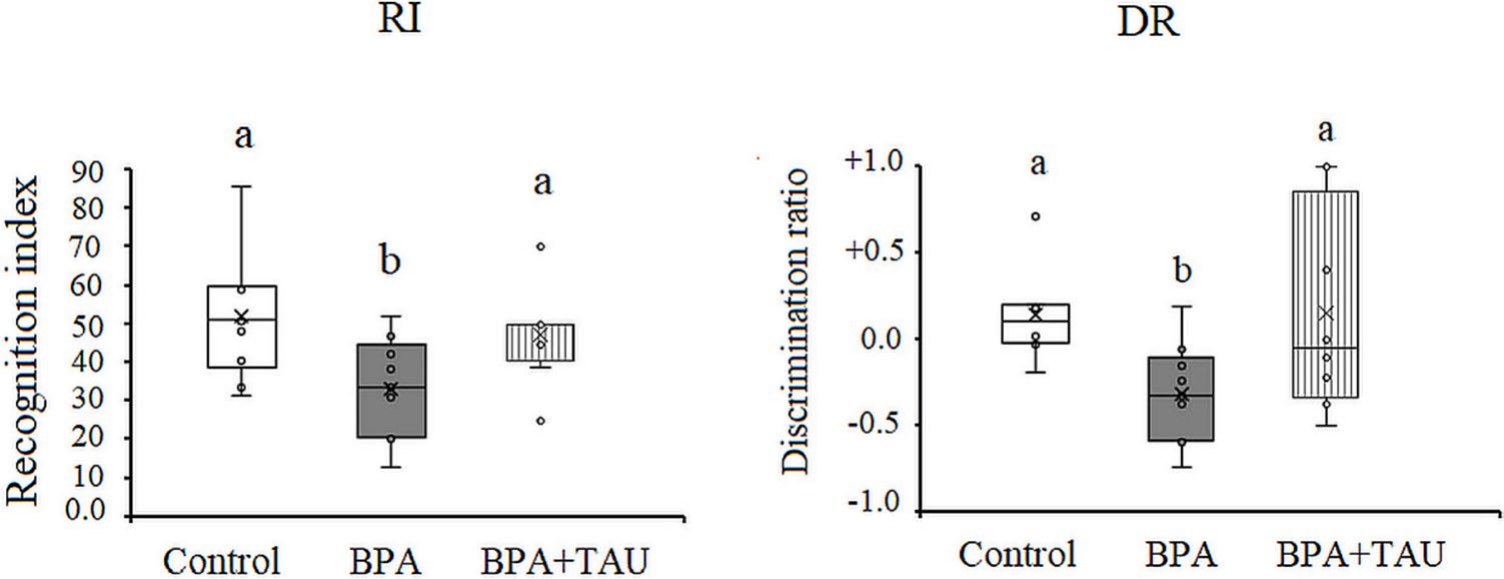
Effect of taurine protection on the recognition index (RI) and discrimination ratio (DR) of novel object recognition test in BPA-treated rats. Different letters indicate a significant change at *p*-value < 0.05, and similar letters mean nonsignificant changes between groups

The treatment of animals with BPA induced a significant decrease (*p* < 0.05) in the RI and DR being − 36.542% and − 345.65% below the control values, respectively. Taurine + BPA-treated animals showed a nonsignificant decline in RI relevant to the control value. However, DR recorded a nonsignificant increase in animals administered with taurine + BPA compared to control animals (Fig. 1).

#### Results of the Y-maze test

**Fig. 2:**
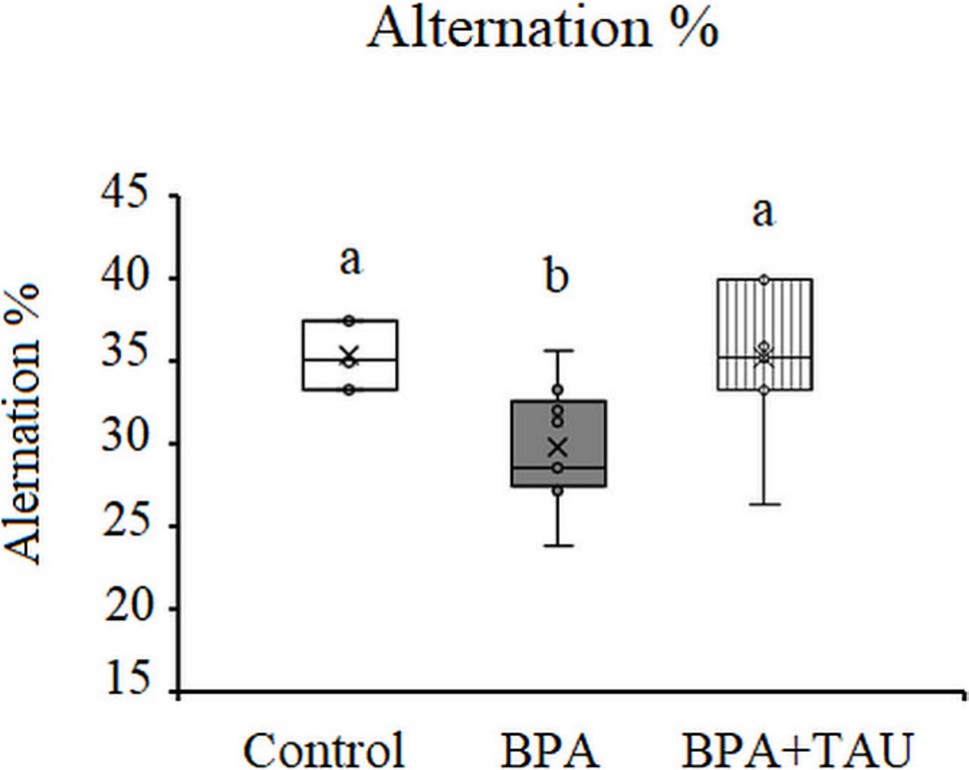
Effect of taurine protection on the spontaneous alternation percent of Y-maze test in BPA-treated rats. Different letters indicate a significant change at *p*-value < 0.05, and similar letters mean nonsignificant changes between groups

Y-maze test showed a significant decrease (*p* < 0.05) in the alternation percent in BPA-injected animals (− 15.02%) compared to the control level. Taurine + BPA-treated animals recorded a nonsignificant increase in alternation percent relevant to the control value (Fig. 2).

#### Results of the open field test

**Fig. 3:**
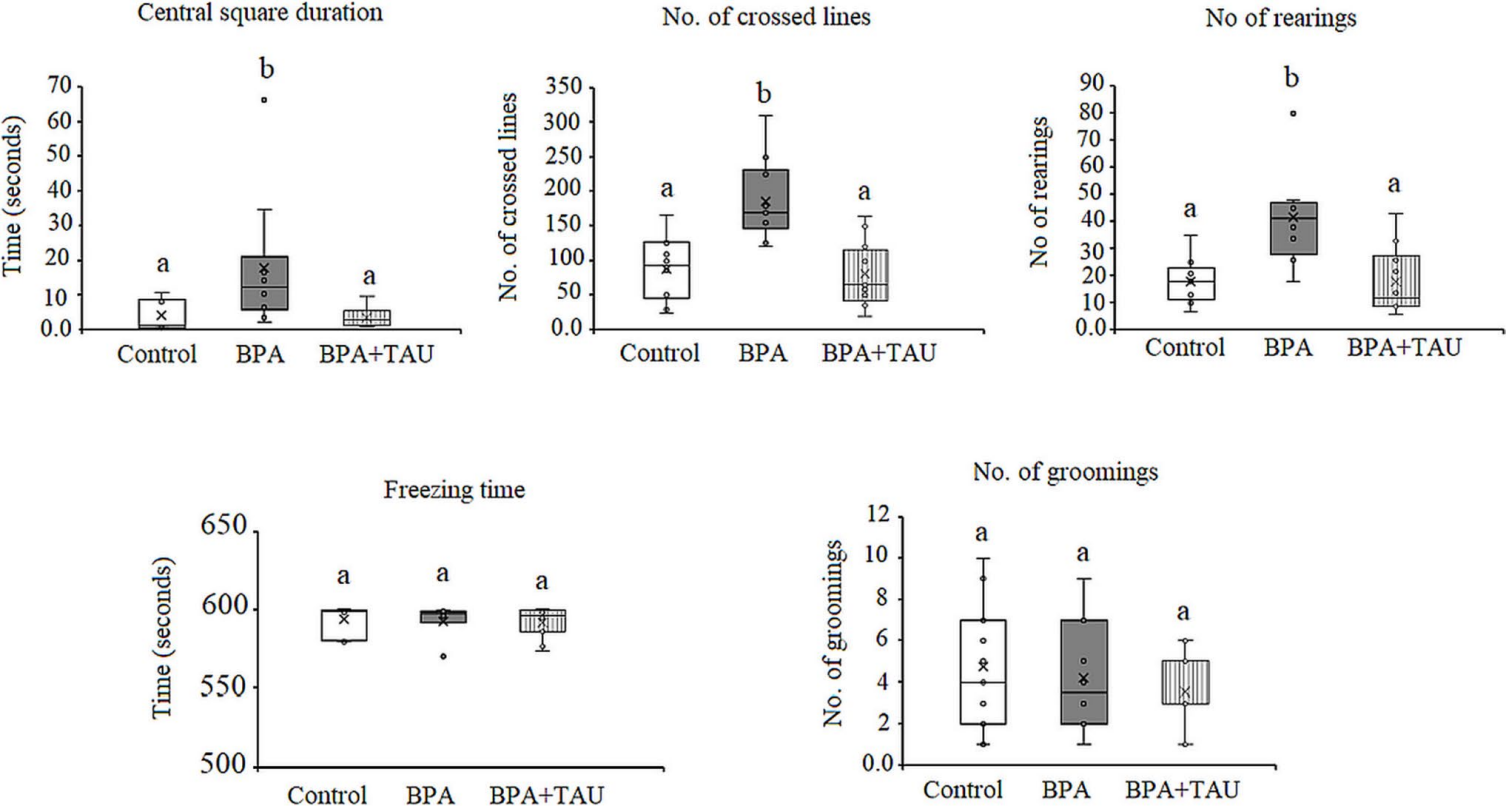
Effect of taurine on the open field test parameters (central square duration, number of crossed lines, number of rearings and freezing time, and number of groomings in BPA-treated rats. Different letters indicate a significant change at *p*-value < 0.05, and similar letters mean nonsignificant changes between groups

Open field data showed a significant increase (*p* < 0.05) in the central square duration, number of crossed lines, and number of rearings in BPA-injected animals being 395.578%, 101.675%, and 109.168% above the control values, respectively. In contrast, BPA-treated animals showed a nonsignificant decline in freezing time relative to control animals. Also, the number of groomings in the BPA model showed no change in comparison to the control group. Taurine + BPA-treated animals revealed a nonsignificant decrease in central square duration, number of crossed lines, freezing time, and number of groomings in comparison with control values. The number of rearings in the taurine + BPA group indicated a nonsignificant increase compared to the control value (Fig. 3).

### Neurochemical data

#### Monoamines data

**Fig. 4:**
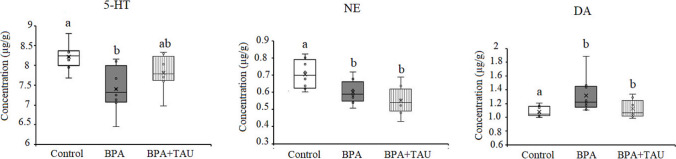
Effect of taurine protection on the levels (µg/g) of serotonin (5-HT), norepinephrine (NE), and dopamine (DA) in the cortex of BPA-treated rats. Different letters indicate a significant change at *p*-value < 0.05, and similar letters mean nonsignificant changes between groups

ANOVA revealed a significant decline (*p* < 0.05) in cortical serotonin and norepinephrine levels in BPA-treated animals being − 9.935% and − 14.87% below the control levels, respectively. On the other hand, dopamine level showed a significant increase (*p* < 0.05) in the BPA-treated group compared to the control level (21.566%). Data in the taurine + BPA group showed a nonsignificant decrease in serotonin level in comparison to the control value. However, a significant decrease in norepinephrine level was obtained in the taurine + BPA group recording − 21.95% below the control value. Meanwhile, the taurine + BPA group revealed a significant increase in dopamine level with regard to the control value (Fig. 4).

**Fig. 5:**
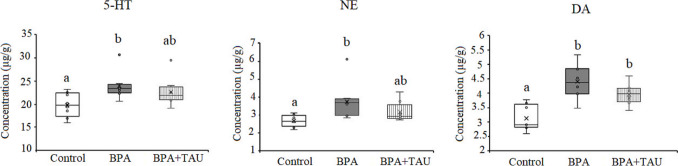
Effect of taurine protection on the levels (µg/g) of serotonin (5-HT), norepinephrine (NE), and dopamine (DA) in the hippocampus of BPA-treated rats. Different letters indicate a significant change at *p*-value < 0.05, and similar letters mean nonsignificant changes between groups

The data of the hippocampus revealed a significant increase (*p* < 0.05) in serotonin, norepinephrine, and dopamine levels in BPA-administered animals recording 20.692%, 40.627%, and 40.970% more than the control levels, respectively. Taurine + BPA-treated animals showed a nonsignificant increment in serotonin and norepinephrine levels in comparison to control values. However, taurine + BPA-treated animals revealed a significant increase (*p* < 0.05) in dopamine level compared to the control value (28.653%) (Fig. 5).

#### Results of enzymes

**Fig. 6:**
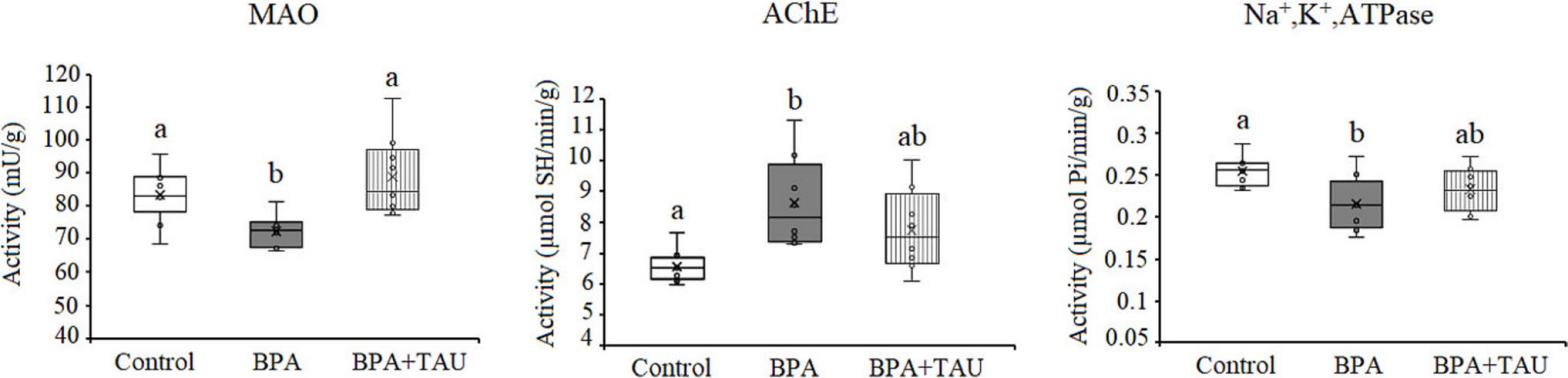
Effect of taurine on the activity of monoamine oxidase (MAO)(mU/g), acetylcholinesterase (AChE) (µmol SH/min/g), and Na^+^,K ^+^,ATPase (µmol Pi/min/g) in the cortex of BPA-treated rats. Different letters indicate a significant change at *p*-value < 0.05, and similar letters mean nonsignificant changes between groups

As shown in Fig. 6, treatment of animals with BPA resulted in a significant decrease (*p* < 0.05) in cortical MAO and Na^+^,K^+^,ATPase activities in comparison to control levels, the percentage differences being − 14.345% and − 15.686%, respectively. On the other hand, AChE activity showed a significant increase (23.700%) in BPA-treated animals compared to control activity. Taurine administration to BPA-treated animals improved the changes in MAO, AChE, and Na^+^,K^+^,ATPase activities in comparison to control values.

**Fig. 7:**
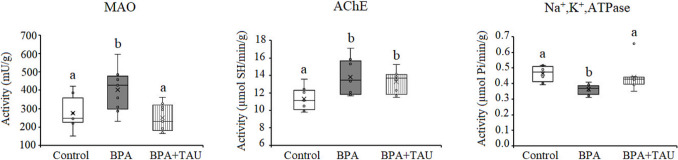
Effect of taurine on the activity of monoamine oxidase (MAO)(mU/g), acetylcholinesterase (AChE) (µmol SH/min/g), and Na^+^,K^+^,ATPase (µmol Pi/min/g) in the hippocampus of BPA-treated rats. Different letters indicate a significant change at *p*-value < 0.05, and similar letters mean nonsignificant changes between groups

In the hippocampus, a significant increase (*p* < 0.05) in MAO and AChE activities was observed in BPA-treated animals in comparison to control values, recording 45.905% and 22.145%, respectively. However, Na^+^,K^+^,ATPase activity decreases significantly by 36.774%. Taurine administration to BPA-treated animals showed a nonsignificant decrease in MAO and Na^+^,K^+^,ATPase activities in comparison to control values. However, administration of taurine to BPA-treated animals increased AChE activity significantly by 17.173% with regard to the control value (Fig. 7).

#### Oxidative stress results

ANOVA test revealed a significant increase (*p* < 0.05) in cortical MDA and GSH levels in BPA-treated animals relevant to control levels, being 27.241% and 31.460%, respectively. On the other hand, the NO level showed a significant decrease (*p* < 0.05) in BPA-treated animals, recording − 26.446% below the control level. Taurine administered to BPA-treated animals resulted in a nonsignificant increase in MDA level (5.191%) and a significant increase in GSH level (35.342%) in comparison to control levels. Moreover, a nonsignificant decrease in NO level was obtained in animals receiving both taurine and BPA in comparison to the control value (Table 1).

**Table 1:** Effect of taurine on the levels of malondialdehyde (MDA) (nmol/g), nitric oxide (NO) (µmol/g), and reduced glutathione (GSH) (mmol/g) in the cortex and hippocampus of rats treated with BPA

		Control	BPA	%D	Taurine + BPA	%D	*p*-value
Cortex	MDA	15.62^a^ ± 1.059	19.87^b^ ± 1.393	27.241	16.43^ab^ ± 1.093	5.191	0.041
	NO	0.12^a^ ± 0.004	0.08^b^ ± 0.006	− 26.446	0.09^ab^ ± 0.010	− 18.181	0.025
	GSH	0.97^a^ ± 0.083	1.28^b^ ± 0.080	31.460	1.32^b^ ± 0.085	35.342	0.016
Hippocampus	MDA	12.21^a^ ± 1.220	18.77^b^ ± 1.837	53.734	16.93^ab^ ± 2.164	38.689	0.036
	NO	0.36^a^ ± 0.021	0.53^b^ ± 0.027	48.342	0.48^b^ ± 0.013	33.977	0.001
	GSH	4.03^a^ ± 0.241	4.99^b^ ± 0.297	23.669	3.88^a^ ± 0.261	− 3.763	0.016

Values represent the mean ± S.E. (*n* = 8)

% D: % difference with respect to control values

Different letters indicate significant changes *p* < 0.05

The same letters indicate nonsignificant changes

In the hippocampus, a significant increase (*p* < 0.05) in MDA, NO, and GSH levels was observed in BPA-injected animals, recording 53.734%, 48.342%, and 23.669% above the control levels, respectively. Taurine + BPA-treated animals showed a nonsignificant increase in MDA level relevant to the control value. In addition, the NO level increased significantly by 33.977% above the control value. However, taurine + BPA-treated animals showed a nonsignificant decrease in the GSH level (Table 1).

#### Caspase-3 results

Table 2 revealed a significant increase (*p* < 0.05) in the cortical and hippocampal caspase-3 levels in BPA-treated animals, recording 745.596% and 742.067%, respectively, compared with the control level. Although taurine administration to BPA-treated animals improved cortical and hippocampal caspase-3 levels by decreasing the percentage differences to 313.210% and 381.971%, respectively, the values were still significantly increased (*p* < 0.05) in comparison to control values.

**Table 2:** Effect of taurine (100 mg/kg) on cortical and hippocampal caspase-3 level in BPA-treated (25 mg/kg) male rats

	Control	BPA	%D	Taurine + BPA	%D	*p*-value
Cortex	**0.70**^**a**^** ± 0.049**	**5.95**^**b**^** ± 0.132**	**745.596**	**2.90**^**c**^** ± 0.060**	**313.210**	**0.001**
Hippocampus	**0.83**^**a**^** ± 0.062**	**7.01**^**b**^** ± 0.091**	**742.067**	**4.01**^**c**^** ± 0.332**	**381.971**	**0.001**

Values represent the mean ± S.E. (*n* = 8)

% D: % difference with respect to control values

Different letters indicate significant changes *p* < 0.05

The same letters indicate nonsignificant changes

### Histopathological data

**Fig. 8:**
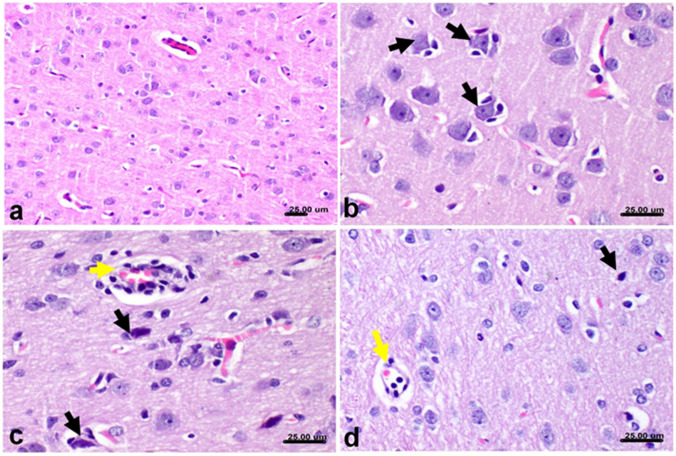
Effect of taurine protection on the histopathological changes induced by BPA in the cortex of rats. Histological section from cerebral cortex stained by H&E. **a** Cerebral cortex grey matter from untreated control group showing a normal histological structure of neurons with normal density of glial cells and blood vessels. **b**, **c** BPA group showing neuronal degeneration with neuronophagia with encircling of necrosed neurons by microglial cells (black arrows) and the reaction involving the perivascular area (yellow arrow). **d** Taurine + BPA group showing individual neuronal degeneration with neuronophagia (black arrow) associated with scarce mononuclear cells in the perivascular area (yellow arrow)

The microscopic examination of the cerebral cortex of the control group revealed the normal histological structure of grey and white matter (Fig. 8a). In the BPA-treated group, the lesions were restricted to the deep cortical layer of cerebral grey matter and were accompanied by multifocal necrosis of large neurons. Neuronophagia and microgliosis (Fig. 8b) were evident with perivascular aggregation of mononuclear cells (Fig. 8c). However, the lesions in the cerebral cortex were extremely reduced in the taurine + BPA-treated group (Fig. 8d).

**Fig. 9:**
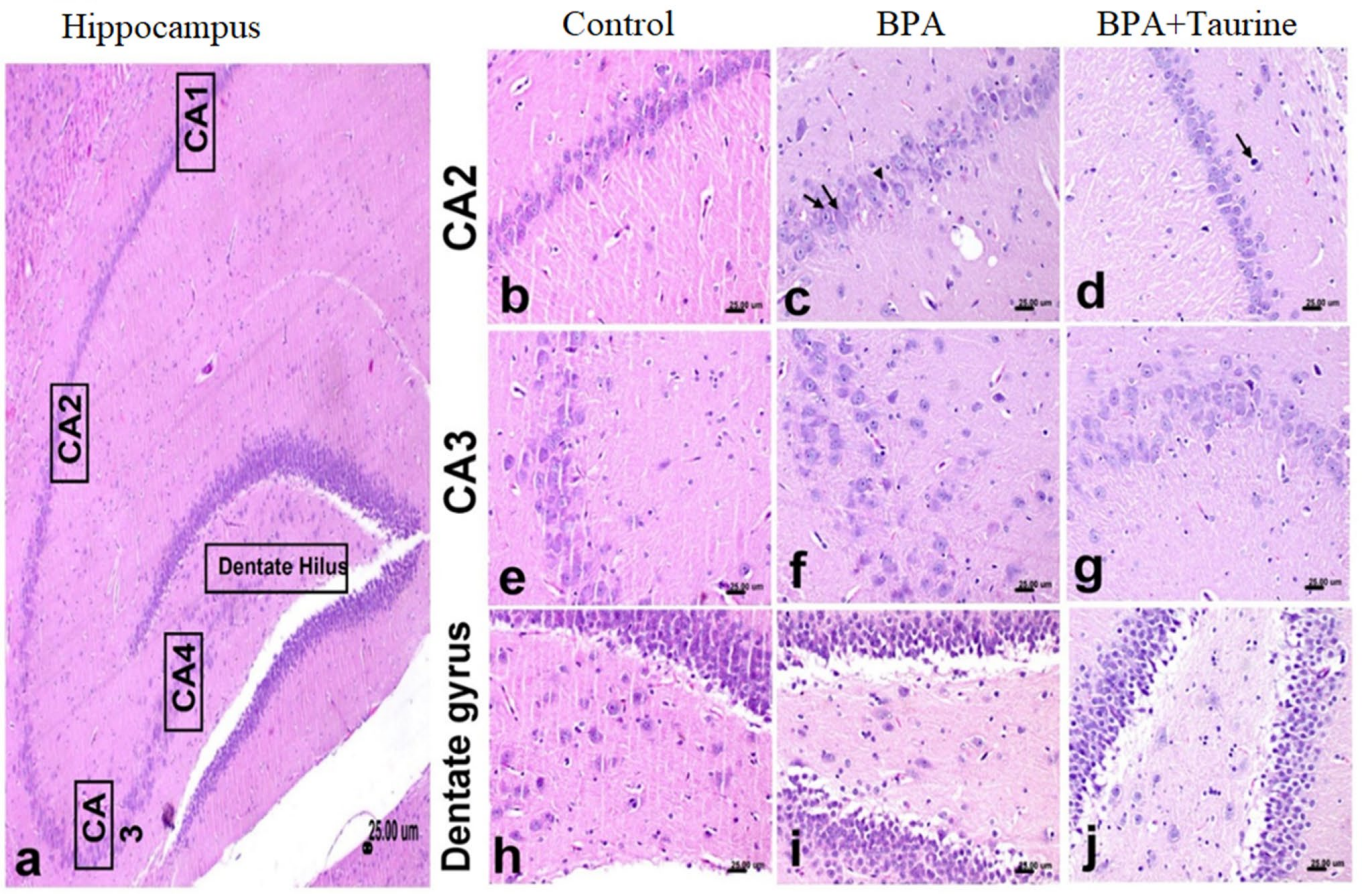
Effect of taurine protection on the histopathological changes induced by BPA in the hippocampus of rats. **a** Histological section from the hippocampus of the control group showing the cellular density comprising different CA areas (CA1–CA4) and dentate gyrus stained by H&E. **b** The normal architecture of pyramidal neurons comprising the CA2 in the control group with normal cellular density. **c** BPA-treated group showing marked necrosis of pyramidal neurons (arrowhead) with chromatolysis of the others (arrow) associated with gliosis and vacuolation of neuropil. **d** Taurine + BPA-treated group showing individual neuronal necrosis (arrow). **e** CA3 from the control group with normal neuronal cellular density. **f** BPA-treated group showing loss of pyramidal neurons with glial reaction. **g** Taurine + BPA-treated group showing focal reduction of neuronal cellular density. **h** Dentate gyrus and hilus from the control group with normal pyramidal cells and glial density of dentate hilus. **i** BPA-treated group showing loss of pyramidal cells with glial reaction. **j** Taurine + BPA-treated group showing gliosis of dentate hilus

The hippocampal areas of the control group are illustrated in Fig. 9a and show a cellular density of neurons comprising different areas of CA (CA1–CA4), dentate gyrus, and hilus. The control group showed normal cellular density of CA1 and CA2 (Fig. 9b). A reduction in the cellular density and necrosis and chromatolysis of pyramidal neurons comprising the CA2 were detected in the BPA-treated group (Fig. 9c), while individual neuronal necrosis was evident in the taurine + BPA-treated group (Fig. 9d). Normal histology of CA3 was detected in control group (Fig. 9e) while severe lesions were detected in CA3 of the BPA-treated group (Fig. 9f) and markedly ameliorated in the taurine + BPA-treated group (Fig. 9g). The dentate gyrus was normal in all experimental groups while the hilus showed normal histology only in the control group (Fig. 9h). Mild focal loss of pyramidal neurons comprising the hilus was detected in the BPA group (Fig. 9i) while gliosis of neuropil comprising the hilus was observed in the taurine + BPA-treated group (Fig. 9j).

## Discussion

Humans are afflicted by continuous exposure to BPA, which leads to the accumulation of this endocrine disruptor throughout life. The lipophilic composition of BPA allows it to penetrate the blood–brain barrier (Hyun et al. 2022) and accumulate in the cerebral tissues (Charisiadis et al. 2018) where it affects the structure and functions of the CNS and influences behavior (Perez-Lobato et al. 2016; Santoro et al. 2019). It has been reported that low levels of BPA could accumulate BPA in adipose tissue as a consequence of long-term oral exposure, leading to toxic effects (Nunez et al. 2001).

Considering the potential nonfood sources of BPA, it is important to note that a significant amount of BPA can be released from resin-based dental materials, estimated at 13 µg and 30 mg of BPA in the average and the worst case scenarios, respectively, after one full crown restoration of a molar (Geens et al. 2012b; Van Landuyt et al. 2011) and that BPA present in thermal papers may be taken in orally through direct contact of unwashed hands with the mouth (Geens et al. 2012b). Thus, in developed countries, human exposure to BPA cannot be avoided due to the massive use of plasticizers in food containers, paints, water, and baby bottles. Therefore, BPA has been detected in human serum, urine, amniotic fluid, samples of placental tissue, and blood drawn from the umbilical cord. BPA exposure can occur orally, topically, and even through inhalation (Ni et al., 2022). Therefore, it was important to investigate the neuroprotective effect of a natural agent such as taurine on the neurochemical, behavioral, and histopathological alterations induced by BPA.

In the Y-maze test, the spontaneous alternation behavior indicates spatial working memory, which is considered a form of short-term memory (Foyet et al. 2011) since rats prefer to investigate the new arm of the maze than enter the same arm they already explored (Kraeuter et al. 2019). The percentage of spontaneous alternation decreased significantly in the present behavioral data, indicating that BPA impaired short-term memory. Moreover, long-term memory was determined by the novel object recognition test, which depends on the innate tendency of rats to discover the new object rather than the old one. The failure of rats to differentiate between the new and old objects is a marker of reduced long-term memory. It is clear from the present data that BPA-induced deficits in long-term memory are indicated by the reduced RI and DR in the novel object recognition test. These results agree with other reports that show that BPA impairs memory (Wu et al. 2020; Tiwari et al. 2024).

It has been reported that the hippocampal and cerebral neurons that are involved in the memory and cognition functions are adversely affected by BPA (Hajszan and Leranth 2010). AChE is the metabolizing enzyme of acetylcholine (ACh), which promotes the transmission of nerve impulses. In addition, elevated central ACh levels can enhance memory and improve CNS function (Huang et al. 2022). However, the increase in brain AChE activity has been shown to induce memory impairments and promote oxidative stress (Melo et al. 2003). Accordingly, the present increase in AChE activity may underlie BPA-impaired memory due to the reduction in cholinergic activity. In addition, the increased AChE activity has been implicated in neuroinflammation mediated by microglial activation (Dasuri et al., 2016). The reduction in Ach, which suppresses glial cells, prevents the release of proinflammatory cytokines such as IL-1β and TNF-α (Xia et al. 2022). Thus, BPA may cause neuroinflammation in the cortex and hippocampus by increasing AChE. An effect may have a role in BPA-induced memory impairment.

The present reduced levels of cortical serotonin and norepinephrine may also contribute to memory impairment induced by BPA. Reduced cortical serotonin level has been implicated in memory deficits (Chakraborty et al. 2019). Norepinephrine contributes to normal cognitive functions in the cortex, including perception, attention, and memory retrieval (Chamberlain and Robbins 2013).

The present data also showed that BPA enhanced motor activity. This was evident from OFT, which is used to measure motor activity, exploratory behavior, and anxiety. OFT showed a significant increase in central square duration, number of rearings, and number of crossed lines in BPA-treated rats. Another behavioral deficit reported by BPA is rat motor hyperactivity during dark and light phases (Kawai et al. 2003; Fan et al. 2018).

Rearing might be a more important indicator of anxiety (Sturman et al. 2018). During rearing, animals stand vertically on the two hind paws in an upright position, which represents an exploratory behavior (Ennaceur 2014). It has been shown that exposure of male and female rats to endocrine-disrupting chemicals exhibited anxiety and depressive-like behaviors (Rebolledo-Solleiro et al. 2020).

In the present study, BPA decreased the cortical levels of 5-HT and NE, which are the substrates of the MAO enzyme. As a result, MAO activity was reduced. This effect could contribute to BPA’s predisposed anxiety behavior. It has been reported that the reduced content of 5-HT in the prefrontal cortex increased anxiety-like behavior (Pum et al. 2009; Yang et al. 2023). Dysregulation in NE participates in the pathophysiology and treatment of depression and anxiety (Goddard et al. 2010).

Serotonin has been shown to inhibit cortical dopamine (Millan et al. 1998). An animal study reported that, during and after aggression, an increase in dopamine levels and a decrease in serotonin levels occurred in the prefrontal cortex (van Erp and Miczek 2000). Therefore, the present increased cortical dopamine level may be caused by the reduced serotonergic activity induced by BPA. The present findings are in parallel with those of Matsuda et al. (2012), who found elevated anxiety-like behavior and dopamine levels in male rats exposed perinatally to BPA.

BPA, as an estrogen partial agonist, has led to the upregulation of the dopamine D1 receptor (Taherianfard and Ahmadijokani 2023). This increase in D1 receptors may be attributed to the increase in dopamine level induced by BPA. This condition of increased dopamine levels and dopamine receptors may exacerbate the adverse effects induced by BPA, such as ADHD and motor hyperactivity. It has been reported that the behavioral alterations induced by BPA in animals are in line with human behaviors resulting from BPA exposure, such as learning deficits, anxiety-like behavior, and hyperactivity (Welch and Mulligan 2022).

The dopaminergic system is concerned with behavioral activation, reward processing, and motivated behavior (Ikemoto and Panksepp 1999). It also participates in the regulation of aggressive behavior. In animal studies, activation of the dopaminergic system is accompanied by increased impulsive aggression (Harrison et al. 1997). Ferrari et al. (2003) suggested that there is an inverse association between dopamine and serotonin levels during aggression. The authors found that aggressive behavior was associated with increased dopaminergic activity and decreased serotonergic activity. Thus, the present decreased 5-HT and the increased dopamine levels in the cortex may underlie the anxiety-like behavior induced by BPA.

In the hippocampus of rats treated with BPA, an increase in dopamine, serotonin, and norepinephrine was observed and was associated with increased MAO activity to meet the increase in its substrates.

NE, DA, and 5-HT play a crucial role in the regulation of different behaviors such as anxiety, exploration, learning, and memory (Myhrer 2003). Therefore, the present changes in monoamine levels in the cortex and hippocampus could explain the behavioral changes caused by BPA.

The present decrease in Na^+^, K^+^,ATPase activity in the cortex and hippocampus by BPA could be attributed to the produced oxidative stress that can inhibit the activity of Na^+^, K^+^-ATPase by oxidation of SH groups and alteration of the membrane fluidity (Silva et al. 2011). The reduced Na^+^,K^+^,ATPase activity has been implicated in memory impairment and causes edema and neuronal cell death (Lima et al. 2008).

Oxidative stress is another factor that may contribute to BPA-induced neurocomplications such as memory impairment (Famitafreshi and Karimian 2018), neurodegeneration (Kim et al. 2015), and motor deficit. Oxidative stress plays an essential role in the pathogenesis of these disorders and is linked to cognitive impairment (Birla et al. 2019). The present data showed that BPA induced oxidative stress in the cortex and hippocampus. This was evident from the increased levels of cortical and hippocampal lipid peroxidation, which is a fundamental constituent of oxidative stress and free radical production. This could be mediated by the impairment effect of BPA on mitochondrial function, leading to mitochondrial dysfunction, overproduction of reactive oxygen species (ROS), neurodegeneration, and cognitive impairments (Agarwal et al. 2016).

Nitric oxide (NO) may act as a free radical, producing nitrosative stress. Reactive nitrogen species increase several toxic molecules and may exacerbate cellular damage in an oxidative stress environment when they are present in high concentrations (Lancaster 2006). It has been reported that BPA exposure stimulates the expression of inducible nitric oxide synthase and neuronal nitric oxide synthase (Wang et al. 2019). This may explain the present elevation in hippocampal NO levels in rats treated with BPA. However, the reduced NO levels in the cortex may be due to its conversion to peroxynitrite, which is a powerful damaging molecule arising from the reaction between NO and superoxide anions. Peroxynitrite stimulates necrosis, apoptosis, autophagy, parthanatos, and necroptosis (Ramdial et al. 2017).

GSH is a major endogenous component of the cellular nonenzymatic antioxidant defense. The present elevated level of cortical and hippocampal GSH may serve as a compensatory counteracting mechanism to combat free radicals produced by BPA. Our previous study has shown a significant elevation in GSH levels as a compensatory mechanism to overcome the oxidative stress resulting from BPA exposure (Khadrawy et al. 2016).

Another factor linked to oxidative stress and correlated with behavioral changes is the present elevated level of caspase-3 in the cortex and hippocampus of animals treated with BPA. This increase could be attributed to the substantial upsurge in caspase-3 expression that was observed in the diencephalon and telencephalon of the brain of zebrafish after chronic BPA exposure (Sahoo et al. 2021). Caspase-3 is the principle enzyme involved in the implementation of apoptosis (Zhang et al. 2013). Cellular redox status may represent the most important factor of the apoptotic pathway that leads to the activation of caspase-3 (Musaogullari et al. 2020). Thus, the increased oxidative stress by BPA may mediate the elevated level of caspase-3 in the cortex and hippocampus. The oxidative stress and the elevated level of caspase-3 may act together to induce cell death. This may in turn explain the present histopathological changes induced by BPA in the cortex and hippocampus.

According to the present results, exposure to BPA as an endocrine disruptor could cause a decline in memory, anxiety, and motor hyperactivity. These behavioral changes were induced due to the elevated AChE activity producing a decline in cholinergic activity and neuroinflammation, increased oxidative stress and caspase-3-induced cell loss, and changes in the monoamine neurotransmitters and Na^+^,K^+^,ATPase activity. These changes resulted in histopathological alterations in the cortex and hippocampus.

Taurine is widely distributed in all animal tissues and performs many biological functions, including osmoregulation, conjugation of bile salt, membrane stabilization, antioxidant, and modulation of calcium and immune activities (Murakami 2015). Thus, taurine was used in the present study as a neuroprotective agent against the changes induced by BPA. In rats, it has been recorded that taurine reached its maximal plasma concentration after 30 min of its oral administration (100 mg/kg) (Nielsen et al., 2017). Therefore, taurine was administered 1 h before BPA to induce its maximal protective effect against BPA whose maximal plasma level was recorded after 1.5 h of its oral administration (Upmeier et al. 2000). Taurine could enter the brain through the taurine transporters (Benrabh et al. 1995). The present behavioral data showed that taurine succeeded in ameliorating memory impairments as indicated by its ability to restore the RI and DR. These data mean that rats regained their long-term memory as they can discriminate between new and old objects. Moreover, the restored percent of alternation indicates that the animal regained their short-term memory and their ability to explore the new arms. Supporting our findings, it was reported that taurine improved cognitive function and protected against neuropathology in an Alzheimer’s disease animal model (Jang et al. 2017).

The present findings also showed that the increased AChE activity induced by BPA was downregulated by taurine. This effect could increase the cholinergic activity that has a substantial role in improving memory and cognition. The attenuated AChE activity could also be beneficial in ameliorating the neuroinflammation induced by BPA. This, in turn, was reflected in the histopathological alterations induced by BPA in the two brain regions that showed a noticeable improvement after taurine treatment. Moreover, the ability of taurine to ameliorate memory may also be linked to its antioxidant activity. Taurine prevented the increase in lipid peroxidation in the two studied brain regions. In addition, the improved NO level by taurine may also reduce nitrosative stress, especially in the cortex.

The antioxidant effect of taurine could be attained by preventing mitochondrial dysfunction (El Idrissi 2008). Taurine is considered a cytoprotective molecule as it has the ability to maintain normal electron transport chain, preserve glutathione stores, and upregulate the antioxidant responses, besides increasing membrane stability and preventing inflammation and calcium accumulation (Baliou et al. 2021).

The present study showed that taurine has an antiapoptotic effect by reducing the increased caspase-3 levels induced by BPA. This effect could be attributed to the success of taurine in reducing caspase-3 expression (Niu et al. 2018). The present findings are supported by the study of Sun et al. (2012), who found that taurine supplementation reduces brain swelling, infarct volume, cell death, and neurological deficits in a stroke rat model.

The present data also showed that taurine restored the number of rearings, the number of crossed lines, and central square time. These results indicate that taurine restored the motor hyperactivity and anxiety induced by BPA. Taurine anxiolytic effect has been reported by Jung and Kim (2019). This effect could be explained by the ability of taurine to improve the changes in serotonin in the cortex and hippocampus. The study of Chen et al. (2019) also reported that taurine reduces hyperactive behavior in SHR rats.

The improved level of serotonin in the cortex and hippocampus and the improved norepinephrine level in the hippocampus in taurine-treated rats were associated with restored monoamine oxidase activity in the two studied brain regions. This improvement may play a role in ameliorating motor hyperactivity and memory deficits induced by BPA. However, the incomplete recovery in catecholamine levels in the cortex and hippocampus, especially dopamine in taurine-protected rats may underlie the incomplete recovery in NO and GSH levels. Dopamine is one of the main sources of oxidative stress during its oxidative metabolism (Li et al. 2020). In addition, dopamine autoxidation is a process that occurred without metabolic enzymes (MAO and catechol-O-methyltransferase). In this process, dopamine interacts with the produced free radicals, forming toxic substances (Miller et al. 1996; Fornstedt Wallin 2024). This may also explain the partial recovery in the level of the apoptotic marker, caspase-3, which showed a significant increase above the control levels in spite of its significant decrease as compared to BPA-treated rats.

The nonsignificant change in Na^+^,K^+^,ATPase activity after taurine administration also contributes to the improvement in memory decline and may be a mechanism by which taurine maintains the osmolarity and homeostasis of the cells.

As a consequence of the antioxidant, anti-inflammatory, and antiapoptotic effects, taurine-treated rats showed improvement in the histopathological changes induced by BPA in the cortex and hippocampus. Reduction in necrosis and lesion, in the present investigation, that has been found in the cortex and hippocampus of taurine-protected animals is parallel to the ameliorative effect of taurine on the caspase 3 level in the protected group.

## Conclusion

In conclusion, taurine exhibits significant neuroprotective effects against BPA-induced cognitive deficits, motor coordination impairments, neurotransmitter imbalances, histopathological alterations, oxidative stress, and apoptosis when administered 1 h before BPA injection. These findings suggest that taurine could have a potential therapeutic effect against the adverse effects of endocrine-disrupting chemicals like BPA on brain function and health. Further studies are recommended to validate the underlying mechanisms and confirm these findings in clinical settings.

## Study limitations

In spite of the importance of the present findings, there are some limitations that should be taken into consideration in future studies. The present study did not take into consideration the potential synergistic effects of BPA with other environmental contaminants, which could collectively influence the present findings. In addition, the level of BPA in serum and in the cortex and hippocampus should be monitored in all groups to exclude the environmental BPA effects. Moreover, the levels of BPA in the cortex and hippocampus should be detected. The study should also investigate the effects of BPA on young in addition to adults.

## Data Availability

All source data for this work (or generated in this study) are available upon reasonable request.
